# Influence of core stability exercise on lumbar vertebral instability in patients presented with chronic low back pain: A randomized clinical trial

**Published:** 2015

**Authors:** Yahya Javadian, Mohammad Akbari, Ghoadamali Talebi, Mohammad Taghipour-Darzi, Naser Janmohammadi

**Affiliations:** 1 Department of Physiotherapy, Babol University of Medical Sciences, Babol, Iran.; 2 Department of Physiotherapy, Iran University of Medical Sciences, Tehran, Iran.; 3 Mobility Impairment Research Center, Babol University of Medical Sciences, Babol, Iran.; 4 Department of Orthopedy, Babol University of Medical Sciences, Babol, Iran.

**Keywords:** Chronic low back pain, Stabilization exercises, Segmental instability

## Abstract

**Background:**

Excessive lumbar vertebrae translation and rotation in sagittal plane has been attributed as an associated factor of lumbar segmental instability (LSI) and low back pain (LBP). Reduction of these abnormalities improves back pain. The aim of this study was to investigate the effect of core stability exercise on the translation and rotation of lumbar vertebrae in sagittal plane in patients with nonspecific chronic LBP (NSCLBP).

**Methods:**

In this randomized clinical trial, 30 patients with NSCLBP due to LSI were included. The participants were randomly divided into two groups of treatment and control. The treatment group received general exercises plus core stability exercise for 8 weeks whereas; the control group received only general exercises. The magnitude of translation (mm) and rotation (deg) of lumbar vertebrae in the sagittal plane was determined by radiography in flexion and extension at baseline and after intervention. The primary outcome measures were to determine the mean changes from baseline in translation and rotation of the lumbar vertebrae in the sagittal plane after 8 weeks of intervention in each group. The secondary outcome was to compare the two groups in regard to translation and rotation of the lumbar vertebrae at the end of the study period. Data were analyzed using paired t-test and independent t-test.

**Results:**

Thirty patients aged 18-40 years old with clinical diagnosis of NSCLBP entered the study. Compared with baseline values, mean value of translation and rotation of the lumbar vertebra reduced significantly in both groups (P<0.05), except L3 translation in the control group. At the endpoint, mean translation value of L4 (P=0.04) and L5 (P=0.001) and rotation of the L5 (P=0.01) in the treatment group was significantly lower than the control group.

**Conclusion:**

These findings indicate that in patients presented with NSCLBP due to lumbar segmental instability, core stability exercises plus general exercises are more efficient than general exercises alone in the improvement of excessive lumbar vertebrae translation and rotation.

Lumbar segmental instability (LSI) defined as aberrant or excessive intervertebral translation and rotational motion, is a cause of nonspecific chronic low back pain (NSCLBP) in 30-35% of patients (1, 2). During lumbar spine flexion-extension motion, some degrees of lumbar vertebrae translation and rotation occur in the sagittal plane which is required for normal kinematic and stability.

The normal values for these movements have been reported as 3-4 millimeters for translation in L1-S1, and 7-13 degrees for rotation in L1-L5 and 14-20 degrees in L5-S1 in sagittal plane (3, 4). In patients with chronic mechanical low back pain, the normal amounts of these movements increase in the involved segments due to dysfunction of motion-controlling elements (5). Local muscles play a significant role in preserving segmental stability and controlling intervertebral motion (6-8). Some researchers reported that impairment in local muscle function may change the extent of segmental vertebral motion. Therefore, local muscle training through specific exercise (stabilization exercise) is expected to improve intervertebral motion and result in better segmental stability (9-11). 

Lumbar core stability exercises are aimed at improving the neuromuscular control, and the endurance of the trunk muscle is necessitated for maintaining spinal stability (12-14). The management of lumbar segmental clinical instability is based on motor control retraining and re-education programs involving postural control retraining, segmental stabilization exercise using transverse abdominis and multifidus co-activation (15-17). The influence of exercise on lumbar spine instability and chronic low back pain has been shown in several studies, but these researches were studied only clinical symptoms (12-17), and up to now, these results were not proved by objective measurements. Therefore, the present study was carried out to investigate the effects of exercise therapy on LSI in patients with NSCLBP for whom the efficacy of treatment was evaluated by radiologic outcomes.

## Methods

In this randomized clinical trial study, 30 patients aged 18 to 40 years with clinical diagnosis of NSCLBP entered the study. Diagnosis of LSI was confirmed by a spine orthopedist according to criteria proposed by Hicks et al. The sensitivity and specificity of this criteria was reported 0.83 (0.61–0.94) and 0.56 (0.40–0.71) respectively (10).

Patients with pregnancy, spinal fractures, herniated discs, acute back pain, systemic disc herniation, osteoarthritis, spondylolisthesis and spondylolysis, lower limb length discrepancy, previous surgery on vertebral column, and other specific diseases causing back pain such as malignancy, rheumatologic conditions, were excluded from the study. A non-probability sampling method was used for grouping.

The outcome measures were recorded before and after treatment by an examiner. The participants and the examiner were blinded for both grouping and treatment method. Informed consent obtained from all participants and the proposal of this study was approved by the Ethics Committee of Medical Sciences. The study was done in Shahid Behesti Hospital in Babol, Iran. 

 The primary outcome measure was the mean changes from baseline in translation and rotation of the lower three lumbar vertebrae in the sagittal plane after 8 weeks of treatment in both groups. The secondary outcome was to compare the two groups in regard to translation and rotation of the lumbar vertebrae at the end of the study period. Changes from baseline were assessed by using computer aided radiographic analysis of spine software (CARA) (18), according to Panjabi's method (19). The validity and reliability of this technique was confirmed in our previous study (17). Distributions of all variables were determined using the Kolmogrov-Smirnov (K-S) test. The relative and absolute reliability were determined via ICC and the standard error measurement. Paired t-test and independent t-test were used to compare quantitative variables and p≤0.05 was considered significant. Data were analyzed with SPSS_16_ statistical software. 

**Measurement of lumbar vertebral translation and rotation in the sagittal plane: **Lumbar vertebral translation and rotation in sagittal plane have been determined using the White and Panjabi's method (19). Putto method was used for taking flexion-extension x-rays images (20) and then, these images were scanned and exported to CARA software environment for measuring the extent of lumbar vertebrae translation and rotation (18). 

**Intervention: **The patients were randomly divided into two groups as control and treatment. The control group performed only a general exercise program (warm up, stretching and strengthening exercise), while the other group performed general exercises plus core stability exercises. Exercises were done for eight weeks, three sessions per week, 60 minutes in each session and each exercise was performed with 10 times repetitions. The exercises progressed gradually from easy to difficult. In addition, the participants performed the exercises three times daily at home which was monitored via phone. In both groups, a light aerobic exercise program including cycling and stretching exercises (totally 15 minutes) were considered for warm up. After warm up, the control group performed some general exercises such as knee-to-chest, bridging, and leg cycling in supine position, heel slides, leg slides and trunk curl. The experimental group in addition to general exercises, received core stability exercises including abdominal hollowing and simultaneous contractions of multifidus and pelvic floor muscles in different positions such as supine, prone, quadruped, bridging, kneeling, sitting and standing. With more progression, the movements of the limbs were added to exercise while the patients were asked to maintain the neutral curvature of the lumbar spine. Swiss balls and balance boards were added to the final level of exercises (21).

## Results

The demographic characteristics of the participants are presented in table1. At baseline, the two groups were comparable (table1). The results showed high intra-tester reliability for the repeated measuring of translation and rotation for all of the segments (ICC=0.70-0.95). Moreover, the standard error of measurement equals to 0.58-1.1 and 3.73-4.45 have been obtained for translation and rotation, respectively. Comparison of patients before and after treatment revealed that the mean of translation and rotation of the lumbar vertebra significantly decreased at the end of intervention in both groups, except for L3 translation in the control group. At the end of the study period, the mean values of translation of the L4 (P=0.04) and L5 (P=0.00) and also rotation of L5 were significantly lower (P=0.00) in experimental group as compared to the control group (table 2).

**Table 1:** Baseline demographic characteristics and t-test for groups matching

**P value**	**Treatment** **Mean±SD**	**Control** **Mean±SD**	
0.277	29.53±6.90	32.93±9.63	Age
0.189	168.60±7.52	172.20±7.11	Height
0.409	68.93±9.25	71.33±6.12	Weight
0.234	6±0.34	5±0.24	LBP Period

**Table 2:** Paired and independent t-test for comparison of translation and rotation of the lower three lumbar vertebrae

	**Control**		**P value** *	**Treatment**		**P value** *	**P value** **
	**Baseline values**	**Endpoint values**		**Baseline values**	**Endpoint values**		
Trans.L3(mm)	2.80±1.59	2.33±1.13	0.13	3.4±0.95	1.68±0.91	0.01	0.19
Trans.L4(mm)	3.74±1.23	2.74±0.64	0.01	3.55±1.16	2.12±.95	0.01	0.04
Trans.L5(mm)	5.29±0.52	4.19±0.64	0.01	5.65±0.6	2.28±0.38	0.01	0.00
Rot.L3(deg)	14.56±1.22	11.56±4.10	0.01	15.18±2.11	13.28±2.89	0.01	0.19
Rot.L4(deg)	16.90±2.27	14.68±3.54	0.03	18.01±1.91	16.28±3.50	0.01	0.22
Rot.L5(deg)	16.70±1.26	14.30±2.04	0.00	17.46±1.21	12.11±2.26	0.01	0.01

mm=millimeter

deg=degree

* P= within groups

** P = between groups

## Discussion

The findings of this study indicated that in patients with NSCLBP due to lumbar segmental instability, core stability exercises in combination with general exercises was more efficient in reducing lumbar instability with respect to general exercises alone. Both methods were effective, but core stability exercises exerted additional benefits which was statistically significant. The results of some studies indicate that core stability exercise results in ameliorates clinical symptoms (13, 14, 17, 21- 28) and functional disability (17) and increase local muscle cross-sectional area (11) and improves the timing and coordination of electromyographic activity (29). However, radiographic studies were not performed in these studies and so the observed beneficial effects were not confirmed radiologically. Several factors such as, increasing local muscle function, co-activation of transverse abdominis and multifidus muscles, and the improvement of lumbar segmental motor control have been attributed to the beneficial effects of exercise (23-25). It is to be noted that the strength of the local muscles was not assessed directly in our study. It is believed that core stability exercises are expected to be effective in patients who have lumbar spine instability at least at one vertebral level (17, 21, 28, 29). The presence of this characteristic in present study may be highlighted the effectiveness of this exercise program. The results of this study should be considered with limitations including low sample size, lack of data collection to determine the effect of exercise on clinical symptoms, absence of follow-up and application of electromyographic measures to assess local muscle strength and activity. Based on our findings, it seems that using of radiographic measurements may be valuable and helpful for the quantitative and objective assessment of lumbar instability. This issue is important because using reliable diagnostic methods can significantly reduce the possibility of placebo effect. The application of radiographic method provides additional supportive data regarding the beneficial effects of core stability exercise in our study.

In conclusion, the results of this study indicate that in patients with NSCLBP, the application of core stability exercises combined with general exercises is more effective than general exercises-alone in improving lumbar segmental instability as documented by radiographic technique. Future studies should determine the correlation between lumbar segment instability and clinical symptoms and evaluate whether the improvement of lumbar instability correlates with clinical improvement.

## Notes

**Funding:** There is no funding involved.
